# Prevalence of Intracranial Artery Stenosis in Patients with Acute Ischemic Stroke in a Tertiary Care Hospital of China

**DOI:** 10.31729/jnma.5201

**Published:** 2020-09-30

**Authors:** Sandip Kumar Jaiswal, Yan Fuling, Min Li

**Affiliations:** 1 Department of Neurology, Affiliated Zhongda Hospital, School of Medicine, Southeast University China; 2 Department of Cardiology, Affiliated Zhongda Hospital, School of Medicine, Southeast University China, China

**Keywords:** *acute ischemic stroke*, *intracranial artery stenosis*, *transcranial Doppler ultrasound*

## Abstract

**Introduction:**

Intracranial artery stenosis is the most common cause of acute ischemic stroke, especially among people in Asia. About its epidemiology, however little is understood. The goal of our research is to establish the prevalence of intracranial artery stenosis in patients with acute ischemic stroke in a tertiary care hospital.

**Methods:**

A descriptive cross-sectional study was done in 1006 acute ischemic stroke patients at Affiliated Zhongda Hospital, School of Medicine, Southeast University China from May 2018 to May 2019. Ethical approval was taken from the Ethical review committee of the institution. A convenient sampling method was done. Intracranial artery stenosis was diagnosed when evidence of acute ischemic stroke was found in the territory of approximately ≥50% stenosis identified by Transcranial Doppler ultrasound and confirmed by magnetic resonance angiography or computed tomography. Statistical analysis was done using the Statistical Package for the Social Sciences version 20.

**Results:**

The prevalence of intracranial artery stenosis was found in 331 (32.90%) patients at 95% Confidence interval (0.24-0.42%). Among 331 cases the anterior circulation artery stenosis was present on 201 (19.98%) patients, followed by posterior circulation artery stenosis on 80 (7.95%) patients, then anterior plus posterior circulation artery stenosis on 50 (4.97%) patients.

**Conclusions:**

Intracranial artery stenosis is one of the most causes of acute ischemic stroke in China. The proportion of anterior circulation artery stenosis was higher than that in the posterior circulation.

## INTRODUCTION

In the global context, stroke is one of the major causes of morbidity and mortality.^1^ Intracranial artery stenosis (ICAS) is commonly associated with acute ischemic stroke (AIS) in Asians, Black, and Hispanic populations, which may account for up to half of ischemic cerebrovascular events in the Chinese populations.^2,3^ However few studies have assessed that the extracranial stenosis is more prevalent in Asia.^4^

ICAS can be diagnosed using various imaging methods. Among these methods, TCD is a safe, non-invasive, and cost-effective diagnostic method to determine ICAS.^5^ The epidemiology of ICAS and its determinants may be more important in Asia than in the USA/Western countries because the stroke has been more prevalent in Asia.^6^ So Early diagnosis is associated with reducing the risk of recurrence stroke.

The goal of our research is to establish the prevalence of ICAS in patients with AIS in a tertiary care hospital.

## METHODS

This is a descriptive cross-sectional study that was performed on 1006 hospitalized AIS patients at the Department of Neurology, Affiliated Zhongda Hospital, School of Medicine, Southeast University China from May 2018 to May 2019. Ethical approval was obtained from the Institutional Review Committee of the institution.

Inclusion criteria included patients hospitalized with clinical features (symptoms and signs) of AIS within 24 hours of onset, patients aged ≥18 years old, and clinical examination and neuroimaging showed AIS. Exclusion criteria included patients with transient ischemic stroke, old infarction, a computed tomography (CT) scan of the head revealed intracerebral hemorrhage, artery stenosis caused by nonatherosclerotic factors such as arteritis, moyamoya disease, and arterial dissection or vasculitis, and cardioembolic stroke.

The sample size was calculated using the following formula:

$$\begin{array}{ll} \text{n} & \text{= }{\text{Z}}^{\text{2}}\times (\text{p}\times \text{q)}/{\text{d}}^{\text{2}}\\ & \text{= }{\left(1.96\right)}^{\text{2}}\times 38\times (62)/{(3\text{)}}^{\text{2}}\\ & \text{= }1005.64 \end{array}$$

Where,

n = sample size
P = prevalence, 38% (educated guess)
q = 1-p
d = margin of error, 3%
Z = 1.96 at 95% CL

Based on the above formula, the minimum sample size at a 95% confidence interval with a 3% error was calculated to be 1005.64 which was rounded to 1006. Convenient sampling was done.

Variables like age, sex, history of hypertension, diabetes mellitus, smoking, dyslipidemia, drinking, coronary artery disease, and history of stroke were obtained from electronic patient records. Hypertension was defined as elevated blood pressure ≥140/ ≥90 mmHg or the need for chronic antihypertensive drugs. Diabetes mellitus has been described as fasting blood glucose ≥7.0 mmol/L, postprandial blood glucose ≥11.1 mmol/L/, or the need for chronic hypoglycemic medication. Smoking has been described as regular cigarette smoking. Hyperlipidemia was described as a total cholesterol level of >220 mg/dl (5.69 mm) or low-density lipoprotein ≥130 mg/dl or the need for a chronic lipid-lowering agent. Drinking was defined as regular alcohol consumption. Coronary artery disease has also been identified in the analysis as risk points. All patients were examined with a CT scan (SOMATOM sensation; Germany) to rule out intracerebral hemorrhage or tumors.

All eligible patients underwent a bedside TCD examination after written informed consent within 6±1.0 hours of admission. All TCD test were performed using a 2 MHz probe (DWL, Munchen, Germany) by an experienced sonographer (with more than 5 years of experience) who was not familiar with the clinical report of the patients by following a standardized rapid (lasting <15 min) insonation protocol.^7^ Ultrasound examination of arteries using TCD is referred to as an insonation.^8^ Patients were examined in a supine position. The transtemporal window was used for the middle cerebral artery (MCA), anterior cerebral artery (ACA), terminal internal carotid artery (TICA), and posterior cerebral artery (PCA) detection and sub-occipital windows were used for the vertebral artery (VA) and basilar artery (BA) detection. At 65-72 mm depth was used for ACA, 50-65 mm for MCA, 65-75 mm for TICA, 65 mm for PCA, 60 to 70 mm for VA, and 85 to 95 mm depth was used for BA insonation. At each point of insonation hemodynamic data such as Peak systolic, mean and end-diastolic velocities, as well as pulsatility index (PSV, MV, EDV, and PI respectively), were noted .

If one of the following features was present, anterior circulation artery stenosis was diagnosed : (1) If the peak systolic velocity (PSV) was greater than 120 centimeters per second at a limited insonation depth, flow stenosis acceleration was diagnosed. To distinguish high-velocity stenosis flow from hemispheric hyperperfusion, only the following conditions were used to diagnose a stenotic lesion : (a) if changes in flow speed were “circumscribed” (maximum changes in flow speed were limited to short segment);(b) if the distal segment flow velocity (ipsilateral terminal internal carotid, middle cerebral, or anterior cerebral artery) were blunted; and (c) if an abnormal spectrum existed (i.e, limited turbulence or musical murmur). (2) If the velocity of the contralateral artery was greater than 30%, the side-to-side difference-stenosis was diagnosed.^9^

Stenosis of posterior circulation artery was diagnosed with usages of maximum PSV or MV obtained from the PCA, VA, and BA with a 4-second spectral Doppler data acquisition sweep. More than ≥50% stenosis was diagnosed when PSV of >100 centimeters per second or MV was more than 60 centimeters per second,^10^ and if the stenotic-to-normal MV ratio was ≥2, with side-to-side differences >20% and circumscribed turbulence or musical murmur at the site of the stenosis.^11,12^

MCA, TICA, ACA, PCA, VA, and BA were classified as mild, moderate, and severe stenosis according to ultrasound criteria and degree of stenosis.^13^ For patients with one of the above arteries having a degree of stenosis ≥50% diagnosed by TCD underwent MRA and/or CTA to varified the degree of stenosis. Digital subtraction angiography (DSA) was only conducted when the result of TCD and MRA/CTA was not fully accepted. The patients, who were not evaluated because of technical issues, were considered as poor windows and the frequency was reported. Extracranial arteries were not checked in this study. Reporting bias from the radiologist can always be present and leads to false data.

Statistical analysis was carried out using Statistical Package for the Social Sciences (SPSS) software, version 20.0 (SPSS Inc, Chicago, IL, USA). Continuous variables were expressed as mean and standard deviation, and categorical variables as numbers (%).

## RESULTS

In the present study, a total of 1107 hospitalized AIS patients participated. Of these 101 patients were inadequate bone windows during TCD examination. Ultimately, therefore, 1006 patients were eligible for analysis. There were 680 (67.5%) males and 326 (32.4%) were female. The mean age of the 1006 patients with AIS was 66.55±10.04 years. All 1006 patients were examined by TCD and confirmed by angiography at least another imaging modality (MRA/CTA or DSA). Hypertension was present in 814 (80.94%) patients and was the most frequently observed risk factors, followed by Diabetes mellitus in 362 (35.98%) patients, 342 (33.99) patients had a history of hyperlipidemia, 331 (32.90) patients were smoker, 181 (17.99) patients had a history of coronary artery disease, 120 (11.92) patients had a history of alcohol consumption, and 251 (24.95) patients had a history of stroke (Table 1).

**Table 1:** Baseline characteristics of the patients with acute ischemic stroke

Variable	n (%)
Age (in years)	66.55±10.04
Gender	
Male	680 (67.59)
Female	326 (32.40)
Hypertension	814 (80.91)
Diabetes mellitus	362 (35.98)
Hyperlipidemia	342 (33.99)
Smoker	331 (32.90)
CAD^*^	181 (17.99)
Alcohol drinker	120 (11.92)
History of stroke	251 (24.95)
Total	418 (41.6)

* CAD = Coronary Artery Disease

The prevalence of ICAS was found to be 331 (32.90%) patients at 95% Confidence interval (0.24-0.42%) at least one artery with a degree of stenosis ≥50%. Patients were categorized according to sex and age group. Out of total female patients, 191 (18.96%) were ICAS and out of total male patients 140, (13.91%) were ICAS.

The samples included were categorized into four subgroups, as follows: 40-49, 50-59, 60-69, and ≥ 70 years. The overall prevalence of ICAS amongst individuals aged ≥ 70 was 230 (22.86%). The prevalence of ICAS in both males and females increased with advancing age. The prevalence of ICAS in female patients was higher than in males except in the age group ≥ 70 years group. None of the male patients with age group 40-49 and 50-59 were ICAS (Table 2).

**Table 2:** Prevalence of intracranial artery stenosis in different age group

Age group	Male n (%)	Female n (%)	Total n (%)
40-49	0 (0)	10 (0.99)	10 (0.99)
50-59	0 (0)	20 (1.98)	20 (1.98)
60-69	20 (1.98)	50 (4.97)	70 (6.95)
≥ 70	120 (11.92)	111 (11.03)	231 (22.95)
Total	140 (13.91)	191 (18.96)	331 (32.90)

All patients were divided into 3 classes according to ICAS severity, which is normal or mild, moderate, and severe ICAS group. The frequencies for each artery and degree of stenosis are listed in (Table 3). MCA had the highest frequencies of moderate stenosis followed by ACA 10 (10) and PCA 7 (7) respectively. No one was found to be a severe ICAS in VA.

**Table 3:** The frequency and the degree of stenosis in different arteries.

Arteries	Normal or mild ICAS n (%)	Moderate ICAS n (%)	Severe ICAS n (%)
MCA^†^	885 (87.97)	110 (10.93)	10 (0.99)
ACA^‡^	895 (88.96)	100 (9.94)	10 (0.99)
TICA^§^	965 (95.92)	30 (2.98)	10 (0.99)
PCA||	965 (95.92)	70 (6.95)	10 (0.99)
BA^¶^	955 (94.93)	40 (3.97)	10 (0.99)
VA^**^	985 (97.91)	20 (1.98)	0 (0)

† MCA = Middle cerebral artery,

‡ ACA = Anterior cerebral artery,

§ TICA = Terminal of internal carotid artery,

|| PCA = Posterior cerebral artery,

¶ BA = Basilar artery,

** VA = Vertebral artery

Our study involved anterior circulation artery stenosis in 201 (19.98%) patients while there were 80 (7.95%) patient's involvement in the posterior circulation artery stenosis, and 50 (4.97%) patients had anterior plus posterior circulation. MCA was the most common involvement of intracranial artery stenosis site 111 (11.04%) followed by ACA 50 (4.97%) and TICA 40 (3.97%) respectively (Table 4).

**Table 4:** Distribution pattern of anterior and posterior circulation artery stenosis.

Location	n (%)
Anterior circulation	201 (19.98)
MCA	111 (11.04)
ACA	50 (4.97)
TICA	40 (3.97)
Posterior circulation	80 (7.95)
PCA	40 (3.97)
BA	20 (1.98)
VA	20 (1.98)
Anterior plus posterior circulation	50 (4.97)
Total	331 (32.80)

†MCA = Middle cerebral artery, ‡ACA = Anterior cerebral artery, §TICA = Terminal of internal carotid artery, ||PCA = Posterior cerebral artery, ¶BA = Basilar artery, **VA = Vertebral artery

## DISCUSSION

The prevalence of ICAS was found out to be 331 (32.90) patients and that the prevalence increased with age. Prevalence of anterior circulation artery stenosis was higher than that of posterior circulation artery stenosis, and MCA was the most prevalent ICAS site. Even in Asian countries the incidence and prevalence of ICAS e varies between the geographical area and ethnicities.^14^ In our finding, the prevalence of ICAS in patients with AIS was found to be similarly noted by Zarei, et al. 29%.^15^ However, the prevalence of ICAS in our study is less than that of Borhani-Haghighi A, et al. 47.46%.^16^ The difference in prevalence is due to different diagnostic criteria used in these studies. For example, compared with the present study, they used to diagnose ICAS with DSA as DSA is the gold standard to diagnose ICAS that reported a higher prevalence of ICAS. The prevalence observed in our study is higher than that of Shariat, et al. 25.4%^17^ in which similar diagnostic criteria were used. Part of the reason for this discrepancy may be because of different time intervals to diagnose ICAS may also lead to a difference in prevalence. For example, TCD was carried out in the previous study at a maximum of 5 days after ischemic stroke. Hence the time interval between identification and TCD may be a key factor in the assessment of TCD's diagnostic accuracy, this can provide enough time for distribution, breakdown, or re-occlusion of thrombus propagation. Discrepancies occur for a long time delay of TCD examination. In our research, the mean TCD exam time period was 6±1.0 hours, which ensured minimal biological changes during this time period.

In our survey, Anterior circulation stenosis was significantly higher than posterior circulation stenosis and MCA was the most common location of ICAS. This finding was similar to the findings of other Asian studies.^18,19^ This can be clarified by anatomical changes in the posterior circulation and for TCD insonation it can be difficult to locate.^20^ Out of the intracranial arteries, the MCA provides blood supply to a wide range of areas and due to its relatively small variation in position and the ease of monitoring with TCD through the temporal window.

In the present study, females were found to be more likely than males to have ICAS, especially in the 40-49, 50-59 and 60-69 year age group. Similar findings were also reported in the previous study.^21^ This difference could be due to the impact of sex hormones.

The research has the strengths that all the TCD examinations are carried out with a standardized protocol by qualified practioners and having a large sample size for a TCD based study. However, there were some limitations to our study. First, there was a single center. Second, due to the insufficient bone window, some patients were excluded. Third, TCD is a technique dependent on the operator that requires considerable knowledge and understanding of intracranial arterial anatomy.

## CONCLUSIONS

In summary, our result showed that ICAS is common in China and anterior circulation artery stenosis is more prevalent than posterior circulation artery stenosis. ICAS was more common in females and in older individuals.

There is high heterogeneity between studies; thus further research is recommended to support these results.
